# The need for COVID-19 research in low- and middle-income countries

**DOI:** 10.1186/s41256-020-00159-y

**Published:** 2020-07-01

**Authors:** Madhu Gupta, Brian Wahl, Binita Adhikari, Naor Bar-Zeev, Sudip Bhandari, Alexandra Coria, Daniel J. Erchick, Nidhi Gupta, Shreya Hariyani, E. Wangeci Kagucia, Japhet Killewo, Rupali Jayant Limaye, Eric D. McCollum, Raghukul Pandey, William S. Pomat, Krishna D. Rao, Mathuram Santosham, Molly Sauer, Rhoda K. Wanyenze, David H. Peters

**Affiliations:** 1 Department of Community Medicine and School of Public Health, Postgraduate Institute of Medical Education and Research, Madhya Marg, Sector 12, Chandigarh, 160012 India; 2 Department of International Health, Johns Hopkins Bloomberg School of Public Health, Baltimore, USA; 3 International Vaccine Access Center, Johns Hopkins Bloomberg School of Public Health, Baltimore, USA; 4 Health Foundation Nepal, Kathmandu, Nepal; 5 Maimonides Medical Center, New York, USA; 6 Epidemiology and Demography Department, KEMRI-Wellcome Trust Research Programme, Kilifi, Kenya; 7 Muhimbili University of Health and Allied Sciences, Dar es Salaam, Tanzania; 8 Eudowood Division of Pediatric Respiratory Sciences, Department of Pediatrics, Johns Hopkins School of Medicine, Baltimore, USA; 9 Papua New Guinea Institute of Medical Research, Goroka, Papua New Guinea; 10 Makerere University School of Public Health, Kampala, Uganda

**Keywords:** COVID-19, Research agenda, Global health

## Abstract

In the early months of the pandemic, most reported cases and deaths due to COVID-19 occurred in high-income countries. However, insufficient testing could have led to an underestimation of true infections in many low- and middle-income countries. As confirmed cases increase, the ultimate impact of the pandemic on individuals and communities in low- and middle-income countries is uncertain. We therefore propose research in three broad areas as urgently needed to inform responses in low- and middle-income countries: transmission patterns of SARS-CoV-2, the clinical characteristics of the disease, and the impact of pandemic prevention and response measures. Answering these questions will require a multidisciplinary approach led by local investigators and in some cases additional resources. Targeted research activities should be done to help mitigate the potential burden of COVID-19 in low- and middle-income countries without diverting the limited human resources, funding, or medical supplies from response activities.

## Background

**Fig. 1:**
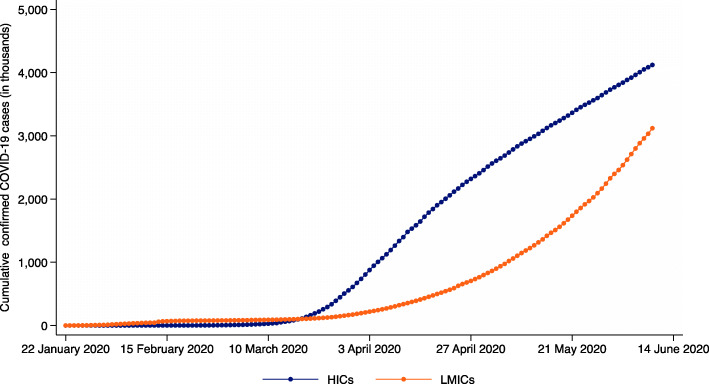
Cumulative cases of confirmed COVID-19 cases in high-income countries and low- and middle-income countries. HICs = high-income countries; LMICs = low- and middle-income countries. Countries categorized based on World Bank classifications. Cases reported from the Diamond Princess and MS Zaandam cruise ships were considered to have occurred in high-income countries. Data from the Johns Hopkins University Center for Systems Science and Engineering (CSSE)

Since late 2019, severe acute respiratory syndrome coronavirus 2 (SARS-CoV-2), the virus that causes COVID-19, has spread throughout the world. Although the pandemic began in China, most reported cases and deaths occurred in high-income countries (HICs) in early months of the pandemic [1]. While it is uncertain how low- and middle-income countries (LMICs) will fare, confirmed cases in these countries continue to increase and could soon overtake confirmed cases in HICs (Fig. 1). We propose three broad research questions to inform public health and policy responses to COVID-19 in LMICs: (1) how do the patterns of SARS-CoV-2 transmission differ in resource-poor settings? (2) how does disease severity in LMICs, particularly among vulnerable populations, differ from observations elsewhere? (3) what will be the impact of pandemic prevention and response measures on the health and wellbeing of the diverse individuals and communities found in LMICs?

## Epidemiology of COVID-19

The transmissibility of infectious diseases, including COVID-19, is driven by characteristics of the causative pathogen, the host (i.e., biological and behavioral factors), and the environment—the so-called epidemiologic triangle. SARS-CoV-2 transmission occurs predominantly within households as compared to diffuse social interactions [2]. Is SARS-CoV-2 transmission different in dense living conditions and larger household sizes common in many LMICs? Demographic profiles are younger in most of Asia and Africa, but crowded multi-generational households are common and therefore could also affect the course of the epidemic. As in many settings, there remain questions related to the primary transmitters of the virus and what characteristics contribute to their infectiousness. Answers to these questions are needed to inform how physical distancing measures and personal hygiene practices can be effectively adapted and implemented in LMIC settings.

Studies have identified SARS-CoV-2 in the feces of infected individuals suggesting possible fecal-oral transmission [3]. Research is needed to understand how long the virus is viable in environments typically found in LMIC settings and if lack of access to clean water and sanitation facilities exacerbates fecal-oral transmission, as has been observed for at least one other respiratory virus (ie, avian influenza) [4]. In a broader sense, does a lack of clean water and soap or hand sanitizer modify transmission through established routes? Emerging evidence also suggests that temperature and absolute humidity lessen transmissibility of SARS-CoV-2; though, this reduction is insufficient to fully disrupt transmission [5]. A more thorough understanding of the relationship between climate, seasonality, and virus transmissibility could provide insights into the potential course of the pandemic in LMICs that tend to be warmer and more humid, supporting preparedness and response efforts in these settings.

## Disease severity

Understanding disease severity in LMICs, particularly case fatality ratios among vulnerable groups, such as young children and immunocompromised individuals, is imperative. COVID-19 causes less severe disease in children compared to adults in HICs with ready access to quality treatment. There are several potential explanations for this [6]. However, the experience with pneumonia—the leading cause of death among children in LMICs and the most common clinical feature of severe COVID-19 cases—raises concerns that COVID-19 might be more severe among children in settings where pediatric respiratory disease risk factors, including multiple severe comorbidities, are more prevalent and advanced treatment for pneumonia is often absent or delayed. In addition, the prevalence of respiratory disease risk factors for all age groups, including undernutrition, indoor and ambient air pollution, being immunocompromised (eg, those infected with human immunodeficiency virus), first and secondhand smoke, and multiple coinfections, is higher in many LMICs. Does the higher prevalence of these risk factors affect COVID-19 disease severity? A more thorough understanding of disease severity and clinical management protocols, particularly in settings with limited laboratory capacity, will help program managers to target prevention and treatment interventions to minimize the impact on vulnerable populations.

## Impact of prevention and control interventions

Understanding the impact and unintended consequences of current COVID-19 prevention and control interventions will help governments and communities to improve and adjust these efforts in real-time. Implementation research can support such efforts. Non-pharmaceutical interventions (NPIs) have been adopted by many LMICs to prevent transmission and reduce morbidity and mortality associated with COVID-19. However, the effectiveness of NPIs in such contexts is not fully understood nor have they been adapted to local contexts. For example, restrictive physical distancing policies have been implemented without regard for the living conditions in dense urban informal settlements or refugee settings. What factors are associated with effective implementation and adherence to these policies in such settings? How do governments and civil society overcome challenges to implementing physical distancing measures in contexts where there is weak governance, low trust in government, inadequate physical infrastructure, and low levels of literacy? How and when should NPIs be relaxed in such settings? Answering these questions will help programs to adapt and improve as the epidemic progresses.

The broader social and economic effects of NPIs in LMICs will also need to be assessed. For example, one major concern with restrictive social distancing measures has been the impact on low socioeconomic households. How will the loss of employment, income, or disruption of food or water affect people’s ability to adhere to lockdown conditions or affect their health and wellbeing? Early modeling suggests that child and maternal mortality could substantially increase as a result of disruptions to routine health services, including immunization, antenatal care, and the provision of family planning services [7]. To what extent has limited transportation, the closure of outpatient services, or other disruptions limited access to essential curative health services, whether for COVID-19 or otherwise? While men appear to have more severe health outcomes associated with COVID-19, current gender norms and gender interactions could contribute to higher risk of infection and differential access to treatment for women [8]. To what extent are these factors exacerbated in LMICs and do they contribute to greater social and economic insecurity for women?

The answers to these questions would provide insights into the effectiveness of NPIs and their social and economic impact. Such information could better support decision-makers in considering the tradeoff between the costs and benefits of mitigation strategies, which would likely differ between LMICs and HICs, while also ensuring protection of vulnerable populations. Further, implementation research should also be used to address priority challenges while the epidemic is ongoing, including: ways to engage communities and the private sector in the response; the utility of different screening approaches for quarantine and isolation, contact tracing, outpatient referrals, and hospitalizations; the provision of crucial health and nutrition services, including services provided by community health workers; and best practices in managing patient flow and clinical case management in particular contexts. These research efforts could serve to strengthen health systems in LMICs once the pandemic has passed.

## Way forward for further research

Answering these pressing questions will require a multidisciplinary effort that includes clinicians, epidemiologists, economists, virologists, behavioral scientists, anthropologists, implementation researchers, and others. The Ebola outbreak in West Africa in 2014 underscored the importance of collaboration across multiple public health and sociological sciences [9]. A “one health” approach to research that also includes environmental, wildlife, and livestock researchers in LMICs will also help prevent or mitigate future pandemics. Where possible, research should be embedded into public health and clinical activities so it does not compete with emergency response activities for limited resources (eg, health workers, personal protective equipment, medical supplies). Data generated from response activities (e.g., contact tracing) can also support research activities without placing an undue burden on response efforts. Robust and innovative information technology systems, including those that use mobile technologies, can facilitate both response and research activities. Conducting research in health emergency settings raises ethical considerations. Independent ethics committees in many LMICs will need to be strengthened to ensure timely review of proposed study protocols. Community-based research will need to be carefully considered and designed to protect research teams, health workers, and community members. This will include protocols to prevent stigma associated with testing positive for COVID-19. In some cases, addressing these research questions will require additional funding. International cooperation here is required, as many LMICs have already allocated substantial resources toward scaling preparedness and response activities. Some bilateral, multilateral, and private funders have already committed to supporting COVID-19 research in LMICs [10]. Research should be initiated and led by local investigators. Too often, LMICs bear the greatest burden of emerging infectious diseases. Rapid, actionable, and locally relevant research could help mitigate the potential burden in LMICs.

## Data Availability

Not applicable.
